# A mutation in the serine protease TMPRSS4 in a novel pediatric neurodegenerative disorder

**DOI:** 10.1186/1750-1172-8-126

**Published:** 2013-08-17

**Authors:** Piya Lahiry, Lemuel Racacho, Jian Wang, John F Robinson, Gregory B Gloor, C Anthony Rupar, Victoria M Siu, Dennis E Bulman, Robert A Hegele

**Affiliations:** 1 Robarts Research Institute, London, ON, Canada; 2 Department of Pediatrics, The Hospital for Sick Children, Toronto, ON, Canada; 3 Departments of Pediatrics, Biochemistry, Microbiology and Immunology, University of Ottawa and Children’s Hospital of Eastern Ontario Research Institute, Ottawa, ON, Canada; 4 Department of Biochemistry, Schulich School of Medicine and Dentistry, University of Western Ontario, London, ON, Canada; 5 Department of Pediatrics and Children’s Health Research Institute, Lawson Health Research Institute, London, ON, Canada; 6 Blackburn Cardiovascular Genetics Laboratory, Robarts Research Institute, 100 Perth Drive, Room 406, London, ON N6A 5K8, Canada

**Keywords:** Autosomal recessive cerebral atrophy (ARCA) syndrome, Neurodegeneration, Trypsin-like serine protease, Homozygosity, Microarray, Exome sequencing, Autosomal recessive inheritance, Old Order Amish

## Abstract

**Background:**

To elucidate the genetic basis of a novel neurodegenerative disorder in an Old Order Amish pedigree by combining homozygosity mapping with exome sequencing.

**Methods and results:**

We identified four individuals with an autosomal recessive condition affecting the central nervous system (CNS). Neuroimaging studies identified progressive global CNS tissue loss presenting early in life, associated with microcephaly, seizures, and psychomotor retardation; based on this, we named the condition Autosomal Recessive Cerebral Atrophy (ARCA). Using two unbiased genetic approaches, homozygosity mapping and exome sequencing, we narrowed the candidate region to chromosome 11q and identified the c.995C > T (p.Thr332Met) mutation in the *TMPRSS4* gene. Sanger sequencing of additional relatives confirmed that the c.995C > T genotype segregates with the ARCA phenotype. Residue Thr332 is conserved across species and among various ethnic groups. The mutation is predicted to be deleterious, most likely due to a protein structure alteration as demonstrated with protein modelling.

**Conclusions:**

This novel disease is the first to demonstrate a neurological role for a transmembrane serine proteases family member. This study demonstrates a proof-of-concept whereby combining exome sequencing with homozygosity mapping can find the genetic cause of a rare disease and acquire better understanding of a poorly described protein in human development.

## Background

Neurodegenerative disorders can be defined as disorders with progressive deterioration of neurological functions such as loss of vision, hearing, and motor function along with previously attained skills [1]. Such degeneration is frequently associated with seizures, feeding issues and intellectual impairment. Neurodegenerative disorders can be etiologically classified under acquired and inherited causes [1]. Acquired causes are most common, and include chronic viral infections, toxin deposition such as heavy metal ingestion and cancer chemotherapy, and drug metabolites [2]. Inherited causes can be due to specific genetic and metabolic defects, such as Rett syndrome, mitochondrial disorders such as MELAS, toxin-generating defects such as PKU, Wilson disease and Hallervorden-Spatz syndrome, lysosomal storage diseases such as Niemann-Pick (types A and C) and Gaucher disease, and leukodystrophies such as Alexander disease [2].

Neurodegeneration in these disorders can involve both the grey and white matter of the brain and spinal cord. Injury of grey matter is irreversible and manifests as seizures, psychomotor retardation, visual impairment and extrapyramidal disturbances such as akathisia (inability to remain motionless) [2]. On the other hand white-matter, mainly represented by myelinated axons, involves disease that is dominated by motor difficulties and chronic encephalopathy [2].

Herein we describe a novel neurodegenerative disorder affecting four infants in a highly consanguineous Amish pedigree. Using homozygosity mapping and exome sequencing, we identified a mutation in TMPRSS4, a transmembrane serine protease, whose physiological role is poorly understood. This work exemplifies the use of unbiased approaches to find novel proteins implicated in rare single-gene disorders.

## Materials and methods

This study had ethics approval by the Office of Research Ethics at the University of Western Ontario (number 07920E). Four participating families from an Old Order Amish community in Ontario provided informed consent and received no financial compensation. All four probands had thorough clinical evaluations, including MRI-head scans. In addition, an autopsy of one proband (V-9) was completed. The pedigrees of the families were assembled through interviews and local Amish community records. In addition, DNA from umbilical cord blood and buccal swabs were extracted from 208 ARCA-unaffected individuals from the Old Order Amish community to determine mutant allele frequency.

Genomic DNA of peripheral blood from 3 affected (V-10, V-12, V-14) and 5 unaffected (V-6, V-8, IV-8, V-15, IV-12) parents and siblings were genotyped using Genome-Wide Human SNP Array 6.0 at London Regional Genomics Centre (http://www.lrgc.ca). Genotypes were called using Affymetrix Genotyping Console, with quality control thresholds for subject call rate (>90%), SNP call rate (95%), Hardy-Weinberg equilibrium (P > 0.0001) and minor allele frequency (>1%). Due to the consanguineous nature of the pedigree, family-based autozygosity or homozygosity mapping was performed using Agilent GT v2.0 software to scan the genotyped SNPs for runs of homozygous blocks. The location scores for each of these homozygous blocks were calculated within Agilent GT v2.0. While a few homozygous block were seen, the one on chromosome 11q clearly had the highest location score and this block was considered to most likely harbour the disease gene [3].

For exome sequencing, targeted enrichment and sequencing were performed on DNA extracted from the peripheral blood of individuals V-10 and V-12, both with typical features of ARCA. Exome enrichment was performed with Agilent SureSelect Human All Exon 50 MB Kit at The Centre for Applied Genomics (http://www.tcag.ca). Paired-end sequencing was performed on Illumina GAII generating 100-bp reads, which were subsequently aligned to the human reference genome sequence (hg19/ GRCh37), using NextGENe software and CLC-Bio Workbench.

*TMPRSS4* in the linked region on chromosome 11q was first screened by Sanger sequencing in an affected individual, a parent, an unaffected sibling, and a non-Amish control. Co-segregation of the *TMPRSS4* variation with disease was demonstrated with direct sequence analysis in a total of 18 family members, including the affected individuals. TaqMan assays were used for identifying allele frequency of the *TMPRSS4* variation in exon 10, c.995C > T (GI:145701031), within 208 Old Order Amish controls and 2382 ethnically diverse and healthy non-Amish controls. TaqMan quantitative real-time PCR assays provided allele discrimination using two allele-specific TaqMan probes synthesized for detecting the *TMPRSS4* variation (allele C: 5′ VIC-CTG CTT CGT AAA GCC and mutant allele A: 5′ FAM-CTG CTT CAT AAA GCC).

Conservation of the TMPRSS4 protein across species was determined with ClustalW, a multiple-sequence-alignment computer program [4]. Impact of the amino acid mutation on TMPRSS4 protein structure, function, and pathological implication was predicted with four online tools, namely PolyPhen-2, Panther, SNPs3D, and PMUT.

## Results

### Clinical characterization of autosomal recessive cerebral atrophy (ARCA)

**Figure 1:**
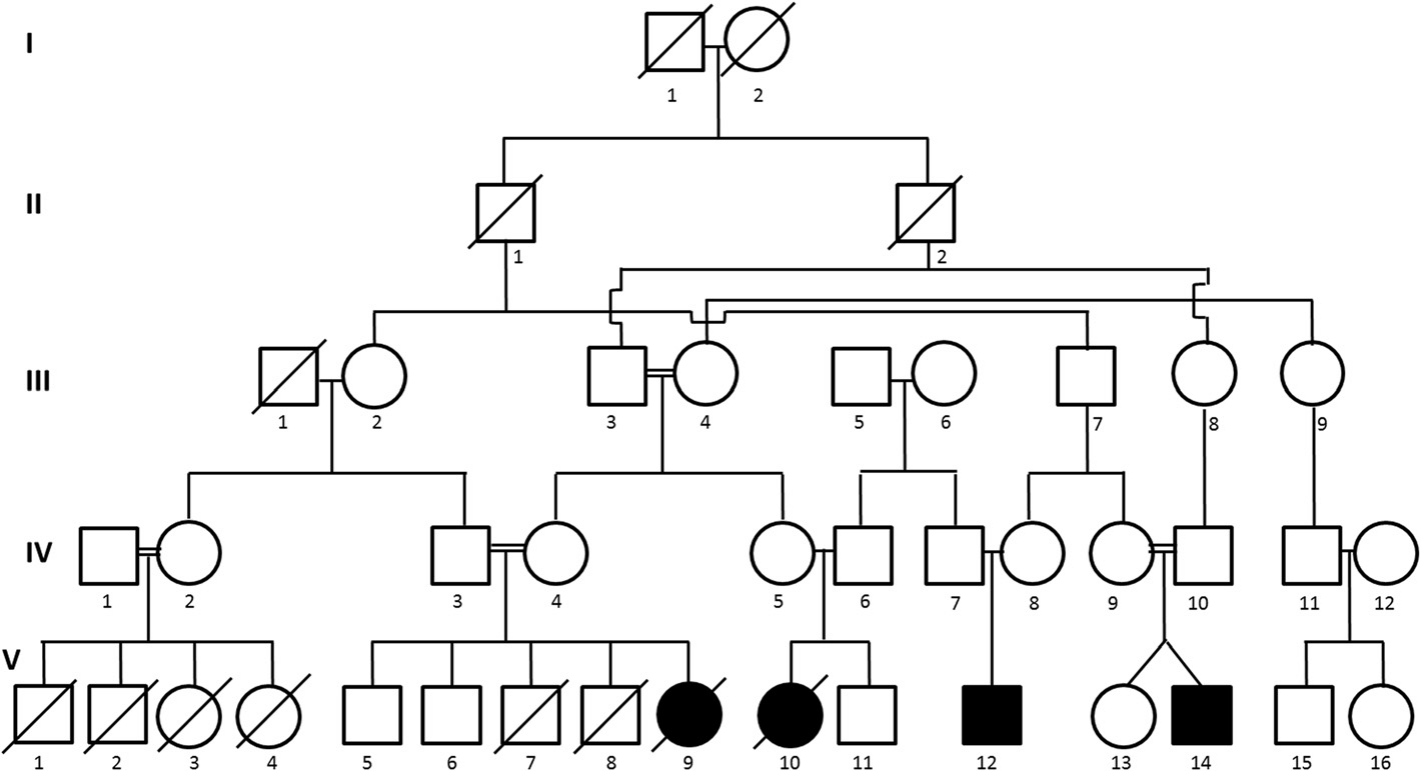
**Old Order Amish pedigree with consanguinity (indicated by the double lines) has four individuals with ARCA, a disease with an autosomal-recessive mode of inheritance.** Affected individuals are shown as blackened squares (male) and circles (female). Diagonal lines across symbols indicate deceased individuals.

Four affected children were identified in an Old Order Amish pedigree (Figure 1) to have similar clinical features and subsequently a thorough clinical evaluation was performed on them (Table 1).

**Table 1:** Clinical description of the four ARCA-affected individuals

		**Affected individuals (year of birth)**			
		**V-9 (2004)**	**V-10 (2000)**	**V-12 (2001)**	**V-14 (1999)**
**Karyotype**		46, XX	46, XX	46, XY	46, XY
**Pregnancy and delivery**		Uncomplicated	Uncomplicated	Uncomplicated	Clomid-induced but uncomplicated
**GA at delivery**		36.5 weeks	38 weeks	39 weeks	39 weeks
**Weight at birth**		2455 g (10th percentile)	2610 g (10th percentile)	N/A	2550 g (<3rd percentile)
**Length at birth**		47.5 cm (25th percentile)	N/A	N/A	N/A
HC, at birth^*^		30 cm (<3rd percentile)	N/A	N/A	N/A
**Fontanelle closure**		at 6.5 mo: Anterior open (N), posterior closed (N)	at 2 mo: Anterior closed (abN)	at 5.5 mo: Anterior closed (abN)	at 8 mo: Anterior fontanelle closed
**Status**		died at 18 months	died at 2 years	alive at 12 yrs	alive at 14 yrs
**Autopsy**		-	+	-	-
**Development milestones**	Suckling	stopped by 6 weeks	stopped by 5 mo	N/A	N/A
	Smile	started at 2 mo, stopped by 7 mo	Started at 2 mo, stopped by 3 mo	no reflexive smile at 1 mo	Started at 2 mo, stopped by 4 mo
	Sitting	unable at 7 mo	unable at 8 mo	unable at 4 yrs	unable at 5 yrs
	Babbling	unable at 7 mo	unable at 8 mo	no speech at 4 yr	N/A
**Neurological signs**	Seizures/Epilepsy	-	+	+	+
	EEG	N/A	marked abnormality with low cortical activity, no epileptiform activity noted	generalized central epileptiform activity	Grade 1 generalized and suppressed cerebral activity
	Spasticity	+ (fisted hands, legs scissoring)	+ (legs scissoring, arms stiff)	+ (myoclonic spasm due to sound/light touch, fisted hands)	+ (clonus of R ankle)
	Reflexes	N/A	brisk, clonus of L ankle	unable to assess	hyperreflexia
	Tone	hypotonic trunk, hypertonic extremities	Hypotonic lower extremities	hypotonic trunk, hypertonic extremities	hypertonic extremities
	Irritable	++	++	+++	+++
	Head circumference, last recorded	36.5 cm @ 6.5 mo (<3rd percentile)	35.5 cm @ 2 yrs (<3rd percentile)	43.3 cm @ 4 yrs (<3rd percentile)	39 cm at 8.5 mo (<3rd percentile)
	Akathisia	+	+	++ (while awake)	++
	Startles	easily	easily	easily however less pronounced presently	easily however less pronounced presently
**Vision and Hearing**	Visual impairment	inconsistent with cues	infrequent, strabismus	cortical, horizontal + vertical nystagmus	cortical visual impairment, eyes roll back
	Fundoscopy	N/A	N/A	mild atrophic fundi	hypoplastic optic nerve and fovea
	Hearing	Normal	normal	normal	normal
**Other organs**	G-tube	-	at 1 yr	at 4 yrs due to recurrent aspiration pneumonitis	-
**Abnormal Bloodwork**		Elevated ammonia, Mn, lactate, platelets		Elevated Mn, lactate, and platelets	Elevated CK, CK-MB, and platelets
**MRI**	Age at time of imaging	10 mo	8 mo, 11 mo,	3 mo, 12 mo	7 mo, 13 mo, 6 yrs 7 mo
	Description	Severe symmetrical cerebral volume loss, gray and white matter affected, thinned corpus callosum. Cerebellum, brainstem, midbrain and supratentorial deep grey matter are unaffected.	Enlarged lateral and 3rd ventricles, severe uniform atrophy of the brain with subcortical white matter loss. Normal spine to T2, cerebellum and pons.	Marked progressive cerebral and cerebellar atrophy with basal ganglia involvement	Prominent ventricles, thin white and grey matter
**CT**	Age at time of imaging	6 mo		3 mo	
	Description	Prominent subarachnoid spaces and ventricular system. Infratentorial compartment is normal, including cerebellum and 4th ventricle		Generalized cerebral atrophy with associated ventriculomegaly, difficult to differentiate between grey/white matter	

* Based on: Barbier et al, 2013. Pediatrics. “New Reference curves for Head Circumference at Birth, by GA”. Abbreviations: N/A, not available; "-", not determined.

V-9, born in 2004, was the product of an uncomplicated pregnancy and delivery at 36.5 weeks gestation, with a head circumference of 30 cm (<3^rd^ percentile). Her neonatal period was unremarkable until 6 weeks of age, when the infant refused to suck. By 3 months of age, she had frequent reflux, was agitated during feeds and was generally irritable. By 7 months of age, she had regressed such that she was unable to smile or laugh, to rollover or sit-up unsupported, and could neither ‘babble’ nor ‘coo’. Growth charts demonstrated progressive microcephaly with head circumference at <3^rd^ percentile at 6.5 months of age. Musculoskeletal examinations demonstrated spasticity (fisted hands and leg scissoring), abnormal tone and constant akathisia with no notable seizure activity. Visual impairment was suggested since she was unable to follow objects by 1 year of age. Investigations ruled out known inborn errors of metabolism and mitochondrial disorders. Of note she had abnormal blood work of unknown significance demonstrating elevated ammonia, manganese, lactate, and platelets levels. MRI and CT scans revealed severe diffuse loss of cerebral volume with symmetrical enlargement of ventricular space, affected grey and white matter with a thinned corpus callosum, however, the cerebellum, brainstem and midbrain were spared. Eventually, she was placed in palliative care since she was unable to swallow or drink independently. She died at 18 months most likely due to failure to thrive, with no follow-up autopsy.

**Figure 2:**
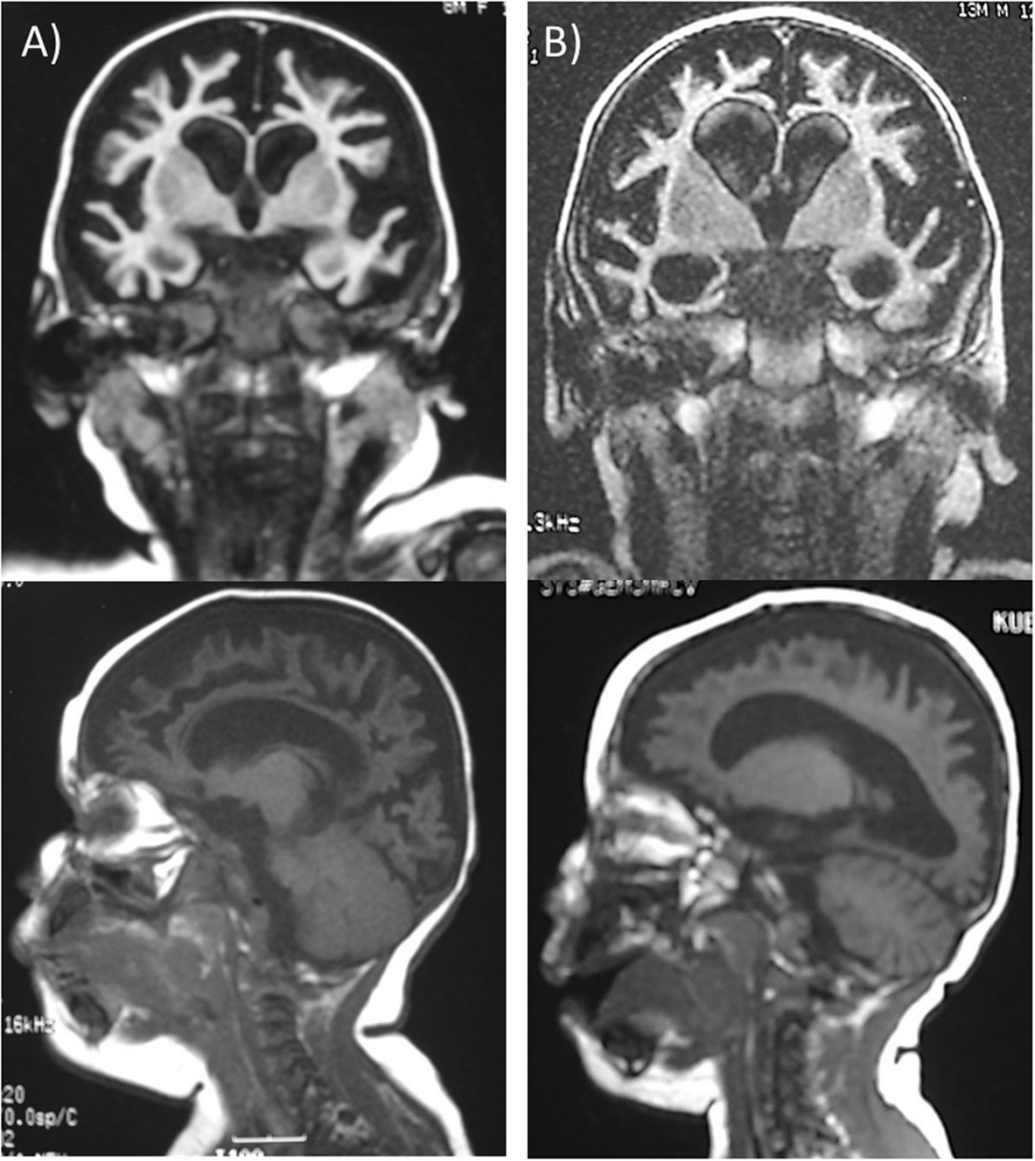
**Coronal and sagittal views of T1-weighted MRI-head of ARCA patients demonstrating diffuse cortical atrophy and ventriculomegaly. A)** V-10 at 8 months of age, **B)** V-14 at 10 months of age.

V-10 was born in 2000 at gestational age of 38 weeks following a normal pregnancy and delivery. Once again, the infant was developing appropriately until 3 months of age when she stopped gaining weight, stopped smiling, was unable to ‘coo’ nor roll over. Suckling regressed by 5 months of age and she had a G-tube insertion by one year of age. Her family doctor noted microcephaly with no head growth post 6 weeks of birth and premature anterior fontanelle closure. At 2 years of age, her head circumference was recorded at 35.5 cm (<3^rd^ percentile). By 6 months of age she developed seizures described to cause head deviation, ipsilateral arm extension, lip biting and a high-pitched scream followed by a brief period of sleep and then arousal. Interestingly, EEG performed at 9 months demonstrated no epileptiform activity. These seizure-like episodes increased in frequency by 15 months of age such that she was placed on a palliative seizure therapy regimen. She also had arm stiffness and leg scissoring indicating spasticity, akathisia, brisk reflexes and hypotonic lower extremities. In addition, she was irritable and startled easily. Investigations ruled out MELAS, inborn errors of metabolism and Hallervorden-Spatz syndrome, and muscle biopsies were normal. MRI demonstrated enlarged lateral and 3^rd^ ventricles with marked atrophy of cortical tissue, sparing the pons and cerebellum (Figure 2A). The infant died at 2 years of age. Although autopsy was unable to prove cause of death, it was concluded that the seizures were not severe enough to cause cortical degeneration. Gross evaluation of brain revealed a pale thalami, normal occipital region, brainstem, and cerebellum as well as symmetrical atrophy of cerebral hemispheres. Microscopic evaluation of the brain demonstrated unaffected brain stem and meninges, material deposition in neurons, like calcium, as well as severe loss of myelin in white matter. The rest of the organs were unremarkable on autopsy.

V-12 was born at 39 weeks gestation in 2001 following an uncomplicated pregnancy and delivery. He had a relatively unremarkable neonatal period except that his parents noted him to have constant akathisia only while awake. He was microcephalic since 3 months of age and was recorded to have a premature anterior fontanelle closure with a head circumference measurement of 43.3 cm at 4 years of age (<3^rd^ percentile). Regression of developmental milestones began within 1 month of age when he stopped smiling, had exaggerated startle response by 3 months; at 4.5 years he was able to kick some toys and bat at objects, but could neither speak nor gesture. He had a diagnosis of cortical visual impairment with atrophic optic fundi and nystagmus by 2 years of age. By 3 months of age, he began having episodes of repetitive extension of arms and hands associated with stiffening of the body with a shriek-like cry with EEG findings of generalized epileptiform activity. By 4 years of age, he was prescribed monthly botox injections and daily oral baclofen, and was wheel-chair bound due to the spasms and abnormal increased muscle tone, which began by 11 months of age. He also had a G-tube insertion to prevent his recurrent aspiration pneumonitis episodes. Investigations ruled out known inborn errors of metabolism and MELAS. Blood work demonstrated elevated manganese, lactate, and platelet levels. Imaging consistently showed cerebral atrophy and ventriculomegaly with subtle atrophy of brainstem and corpus callosum. At 12 years of age, he continues to live with his family and is fully dependent on others, however his seizures are better controlled seizures and he is less irritable.

V-14 was the first known proband with ARCA in this pedigree born in 1999. He was a product of a clomid-induced twin pregnancy with a fraternal sister who continues to have normal development. Delivery was induced at 39 weeks with a birth weight of 2550 grams (<3^rd^ percentile). His neonatal period was unremarkable overall. He was microcephalic prior to development regressions, which were first noted after 4 months of age. By 4 months he stopped smiling, laughing, cooing, and had abnormal tone. By 9 months of age, he would be easily startled, irritable, and agitated while feeding. By 5 years, he could only hold his head up, was severely spastic, had hyperreflexive extremities, and was unable to eat solid foods. By 3 years of age he was diagnosed with visual impairment due to cortical abnormalities, with hypoplastic optic nerve and fovea. Investigations demonstrated no lysosomal, peroxisomal or metabolic disorders. EEG demonstrated supressed Grade 1 cerebral activity with generalized abnormal cortical activity. MRI of spine demonstrated no abnormalities; however, ventricles and sulci were prominent in size and severely atrophic white and gray matter (Figure 2B). He is presently 14 years old and continues with generalized spasticity and requires a pureed diet, which he regularly chokes on. He is less irritable and on no medications.

Overall, all four children were products of an unremarkable pregnancy, delivery and neonatal period. However, acquired microcephaly was noted within 2 months of birth. By 4 months of age, the infants displayed constant irritability, regression of developmental milestones, akathisia, increased startle response, spasticity, abnormal tone, visual impairment and seizures. In two of the four children, the anterior fontanelle had prematurely closed. Brain imaging of all four affected children revealed a characteristic ventriculomegaly and progressive symmetrical atrophy of brain matter—in particular the white and grey matter of the cortex, sparing the infratentorial segments, such as the midbrain, brainstem and cerebellum.

Based on the clinical findings and imaging, we named this novel disorder as Autosomal Recessive Cerebral Atrophy (ARCA).

### Molecular investigations

**Figure 3:**
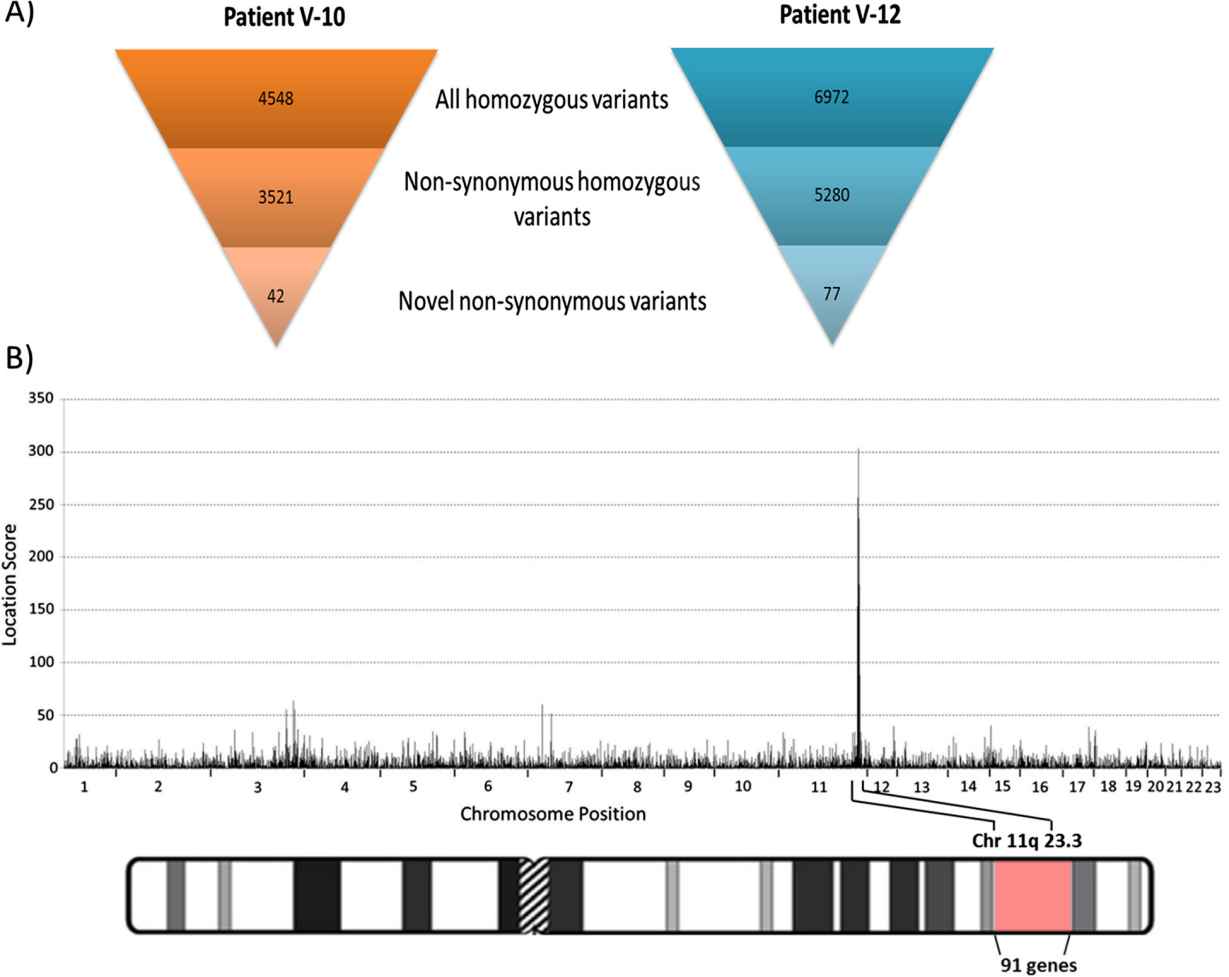
**Genetic investigations to find the causative mutation for ARCA. A)** Variant filtration of exome sequencing data of two affected individuals, V-10 and V-12. Non-synonymous is defined as a change that alters the amino acid. **B)** Homozygosity mapping narrows candidate region to 11q23.3, containing 91 genes.

**Figure 4:**
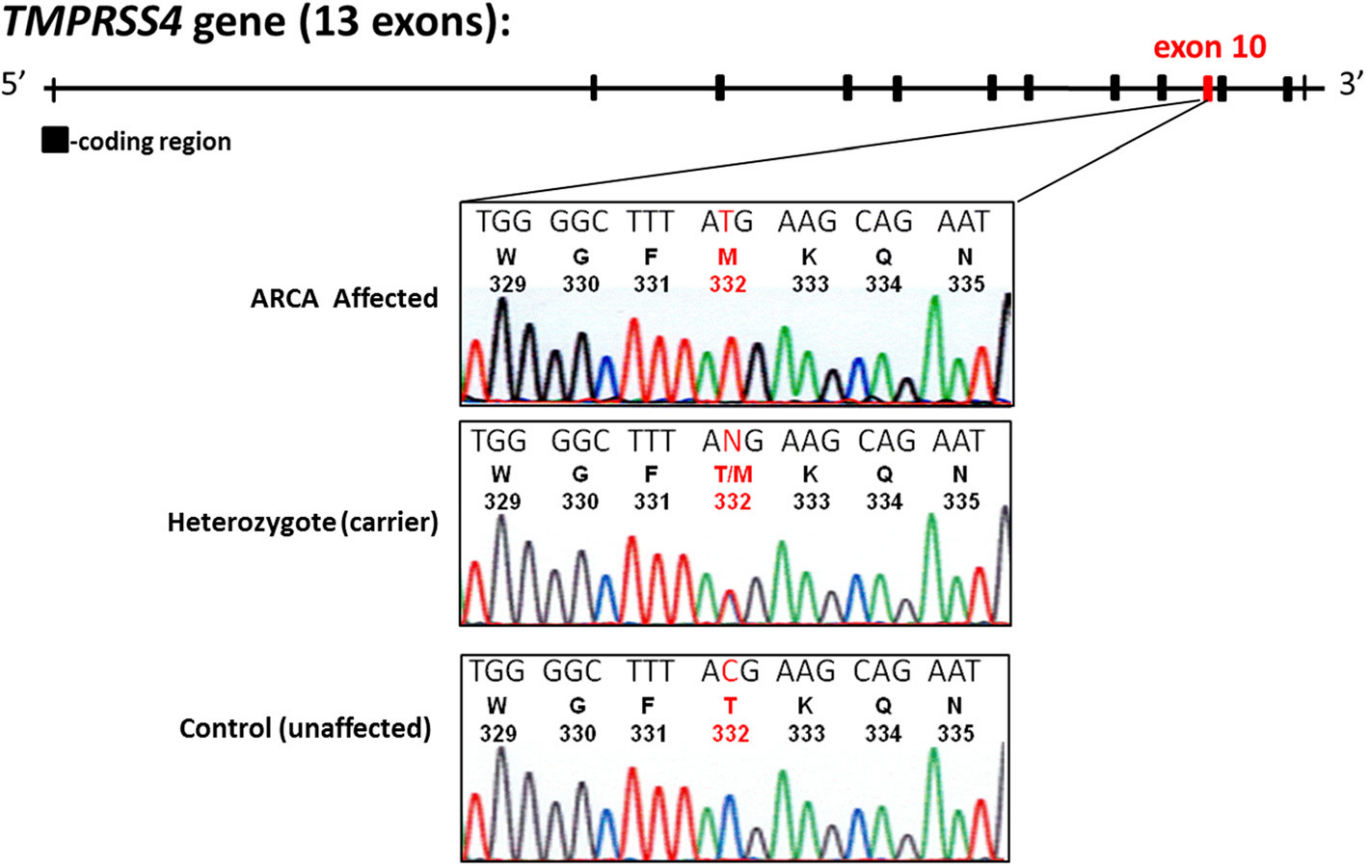
**The genomic structure of *TMPRSS4 *gene consists of 13 coding exons with a nucleotide change in exon 10 (indicated in red) that alters threonine to methionine at residue 332 (T332M).** DNA sequence tracings of *TMPRSS4* exon 10 in an ARCA affected (top), T332M heterozygote (middle), and an unaffected (bottom) individual. For each tracing, the nucleotide sequence is shown at the top followed by the single-letter amino acid code and codon numbers beneath.

Exome sequencing was performed on 2 affected individuals (V-10 and V-12) and a filtering method was applied to identify unique nucleotide variants, which yielded 42 and 77 novel homozygous sequence variants in individuals V-10 and V-12, respectively (Figure 3A). Homozygosity mapping revealed a candidate region, the homozygous block with the highest location score (Figure 3B), encompassing 4.5 Mbp in chromosome 11q with a location score of 303 comprising of 872 SNPs and 91 genes (Figure 3B). Within this candidate region in chromosome 11, only 1 of the unique nucleotide variants was present in homozygous state in both affected individuals while their parents were obligate heterozygotes. The variant is a C to T at c.995 in exon 10 of the *TMPRSS4* gene, resulting in an amino acid change from threonine (T) to methionine (M) at residue 332 (T332M, Figure 4). Sanger sequencing of *TMPRSS4* c.995C > T in the pedigree is consistent with an autosomal recessive inheritance pattern, and segregates with the ARCA phenotype (see Additional file 1). The frequency of the T allele was assessed within 208 Old Order Amish community members (excluding the pedigree). None had the TT genotype while 12% had the CT genotype (carrier rate); thus 1 in 250 Old Order Amish are predicted to have the TT genotype. In 2382 geographically and ethnically diverse controls, all had the CC genotype suggesting that the TT genotype is a private mutation within this Old Order Amish community.

### *In silico* investigations

**Figure 5:**
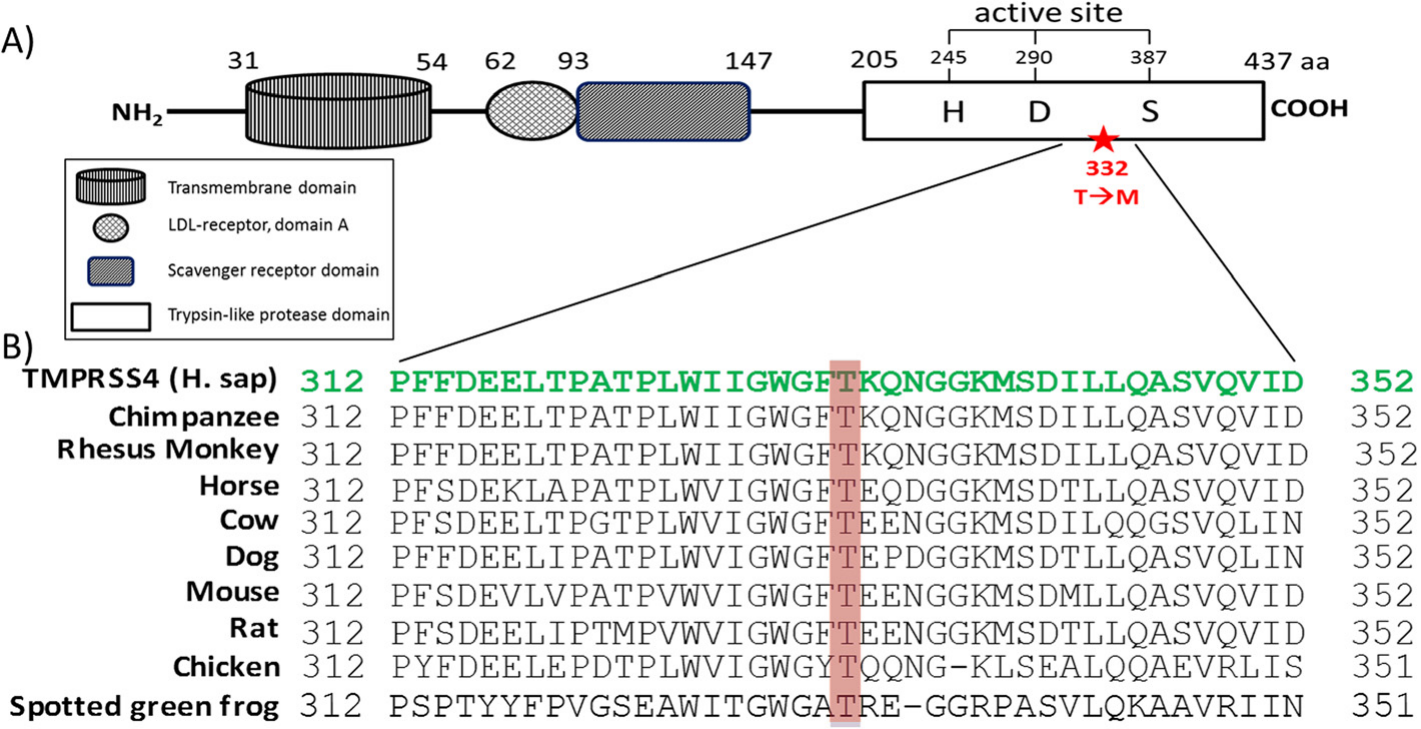
**TMPRSS4 protein structure and amino acid sequence alignment with its orthologs. A)** A schematic representation of TMPRSS4 protein domain structure. At the N-terminal the protein has a transmembrane domain, a LDL-receptor A domain, and a scavenger receptor domain. The C-terminal encodes for the trypsin-like serine protease domain which contains the catalytic triad amino acids (histidine, aspartic acid, serine). The T332 residue (indicated with a red star) is also found within this domain. **B)** Multiple alignments (using ClustalW) demonstrate that T332 residue (highlighted in red) is highly conserved across a representative set of species-specific TMPRSS4 orthologs.

To better understand the biological significance of this mutation, various bioinformatics strategies were employed. Residue 332 is within the trypsin-like serine protease domain (Figure 5A). The ClustalW protein sequence alignment program showed strong conservation of this residue among TMPRSS4 orthologs from other organisms (Figure 5B). PolyPhen, Panther and SNPs3D, similarly showed that the T to M change at residue 332 would be deleterious or damaging to TMPRSS4 function.

The crystal structure of TMPRSS4 has yet to be published and there is no related structure with an identity >40%. However, examination of the domain structure of TMPRSS4 shows that the C-terminal half of the protein is an excellent match (E-value of 3.1e-91) to the trypsin-like serine protease family (cd00190) [5]. Alignment of the TMPRSS4 sequence with the available structures of this family, using the Cn3D tool [6], demonstrate that residue 332 is in a conserved loop that borders the active site. In majority of these homologous structures, the residue side chain is partially exposed on the surface and in one structure (1MKX) the side chain is directed into the active site. In either case, the T332M change—a polar to a non-polar amino acid coupled with a longer side chain—would likely result in a steric clash that could lead to substrate binding alteration and thus altered TMPRSS4 function.

## Discussion and conclusion

We describe a novel pediatric disorder in an Old Order Amish community, named Autosomal Recessive Cerebral Atrophy (ARCA) due to a progressive brain atrophy leading to neurodegenerative symptoms including loss of developmental milestones, seizures and loss of motor function.

Although there was slight variability in the age of developmental milestone regression, by 4 months of age all 4 infants displayed irritability, akathisia, increased startle response, abnormal reflex and tone, visual impairment, as well as seizures or seizure-like episodes. In addition, the infants were noted to have microcephaly early in life and brain imaging revealed a characteristic ventriculomegaly and progressive symmetrical atrophy of brain matter, particularly cortical white and grey matter. Even though the degenerative process of the brain matter most likely started earlier in development, the infants may have been asymptomatic perinatally or during the neonatal stage, since the cerebrum is functionally silent during the early weeks of life [7]. It is likely that 2 of the 4 children continue to survive due to the level of medical intervention provided by the caregivers rather than biological variability in the severity of the disease.

After conducting a comprehensive literature search of the clinical descriptions of ARCA, we identified three similar yet distinct syndromes. For instance, patients with micropthalmia, brain atrophy (MOBA; MIM: 611222), develop extensive atrophy of white and grey matter after 6 months of life leading to progressive spasticity and seizures. However, unlike ARCA, MOBA patients also have congenital ocular malformations [8]. A case report, in 2011, describes two Japanese sisters with failure of postnatal brain growth with similar brain MRI findings to those of ARCA-affected patients. However, these sisters also had dysmorphic facial features and simplified gyri that were not present in ARCA [9]. Furthermore, West syndrome or Early Infantile Epileptic Encephalopathy 5 (EIEE5, MIM: 613477) is similar to ARCA in that the seizures manifest around 3 months of age due to brain atrophy [10]. However this atrophy is mostly localized to the brainstem due to hypomyelination of white matter in this region. EIEE5 is due to a heterozygous mutation in *SPTAN1* gene encoding spectrin, a cytoskeletal protein involved in scaffolding [11].

Using exome sequencing and homozygosity mapping we identified a homozygous missense (T332M) mutation in the serine protease, TMPRSS4. This mutation segregated with the disease and no carriers were found outside of the Amish community, giving genotypic and statistical evidence that the *TMPRSS4* mutation is likely causative of ARCA. Bioinformatics further illustrated that this T to M mutation at residue 332, most likely affects protein function due to: 1) high evolutionary conservation of the residue, 2) program predictions that demonstrate pathological consequence, and 3) predicted changes in protein structure leading to instability.

Serine proteases, such as trypsin-like proteases are ubiquitously distributed and play pivotal roles in the gastrointestinal, reproductive, and immune systems [12]. Trypsin-like serine-proteases have been implicated in brain plasticity, neural development, neurodegeneration and neuroregeneration through human disorders and knock-out zebra fish models [13]. Specifically, one of the first serine proteases shown to be involved in human non-syndromic mental retardation was neurotrypsin [14]. Of note, the family members of Type II transmembrane serine proteases (TMPRSSs), TMPRSS2, TMPRSS1, TMPRSS3, and TMPRSS5 have only been implicated in certain types of sensorineural deafness [15]. TMPRSS4 is expressed in the gastrointestinal tract, skeletal muscle and brain. It is highly expressed on the cell surface of pancreatic, thyroid, lung, and other cancer tissues, with potential biological implication in cell invasion and migration [16].

This study demonstrates that TMPRSS4 is strongly implicated in the causation of this autosomal recessively inherited pediatric neurological disease. This information can now be used for genetic counselling in the community and to stimulate mechanistic research on this protein and mutation.

## Abbreviations

ARCA: Autosomal recessive cerebral atrophy; TMPRSS4: Transmembrane protease, serine 4; SNP: Single nucleotide polymorphism; G-tube: Gastrostomy tube; MELAS: Mitochondrial encephalomyopathy, lactic acidosis, and stroke-like episodes; EEG: Electroencephalogram.

## Competing interests

The authors declare that they have no competing interests.

## Authors’ contributions

PL and RAH were involved in design, acquisition and analysis of data, and drafting of the manuscript. JW and JR were in involved acquisition and analysis of data.LR, GBG, CAR, VMS, and DEB were involved in design, acquisition and analysis of data, and made contributions to the draft of the manuscript.

## Supplementary Material

Additional file 1 **Figure of the pedigree illustrating ARCA phenotype along with genotype data Figure legend (description)—Sanger sequencing of *TMPRSS4 *c.995C>T in the pedigree is consistent with an autosomal recessive inheritance pattern and segregates with the ARCA phenotype.** The top half of each symbol indicates whether the individual is ARCA affected (black) or unaffected (white). The bottom half of the symbol represents the genotype with T shown in black and C shown in white. Click here for file
