# Supplementary figures and images for: A mutation in the serine protease TMPRSS4 in a novel pediatric neurodegenerative disorder
Source: Orphanet J Rare Dis. 2013 Aug 17;8:126. doi: 10.1186/1750-1172-8-126 (PMC3765793; doi:10.1186/1750-1172-8-126)

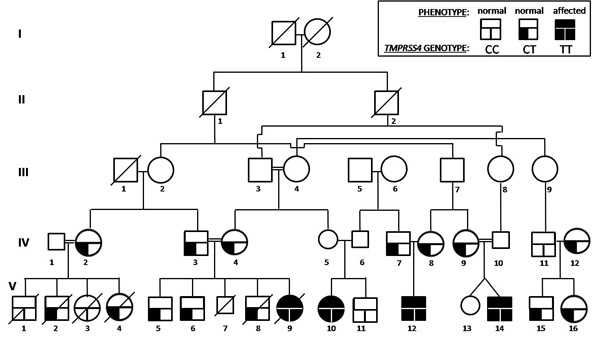

Supplement: Additional file 1 — Figure of the pedigree illustrating ARCA phenotype along with genotype data Figure legend (description)—Sanger sequencing of TMPRSS4 c.995C>T in the pedigree is consistent with an autosomal recessive inheritance pattern and segregates with the ARCA phenotype. The top half of each symbol indicates whether the individual is ARCA affected (black) or unaffected (white). The bottom half of the symbol represents the genotype with T shown in black and C shown in white. [file 1750-1172-8-126-S1.jpeg]
